# Better medicine through machine learning: What’s real, and what’s artificial?

**DOI:** 10.1371/journal.pmed.1002721

**Published:** 2018-12-31

**Authors:** Suchi Saria, Atul Butte, Aziz Sheikh

**Affiliations:** 1 Machine Learning and Healthcare Laboratory, Departments of Computer Science, Statistics, and Health Policy, Malone Center for Engineering in Healthcare, and Armstrong Institute for Patient Safety and Quality, Johns Hopkins University, Baltimore, Maryland, United States of America; 2 Bakar Computational Health Sciences Institute, University of California, San Francisco, San Francisco, California, United States of America; 3 Center for Data-Driven Insights and Innovation, University of California Health, Oakland, California, United States of America; 4 Usher Institute of Population Health and Informatics, The University of Edinburgh, Edinburgh, United Kingdom

## Abstract

Machine Learning Special Issue Guest Editors Suchi Saria, Atul Butte, and Aziz Sheikh cut through the hyperbole with an accessible and accurate portrayal of the forefront of machine learning in clinical translation.

Artificial intelligence (AI) as a field emerged in the 1960s when practitioners across the engineering and cognitive sciences began to study how to develop computational technologies that, like people, can perform tasks such as sensing, learning, reasoning, and taking action. Early AI systems relied heavily on expert-derived rules for replicating how people would approach these tasks. Machine learning (ML), a subfield of AI, emerged as research began to leverage numerical techniques integrating principles from computing, optimization, and statistics to automatically “learn” programs for performing these tasks by processing data: hence the recent interest in “big data.”

Although progress in AI has been uneven, significant advances in the present decade have led to a proliferation of technologies that substantially impact our everyday lives: computer vision and planning are driving the gaming and transportation industries; speech processing is making conversational applications practical on our phones; and natural language processing, knowledge representation, and reasoning have enabled a machine to beat the Jeopardy and Go champions and are bringing new power to web searches [1].

Simultaneously, however, advertising hyperbole has led to skepticism and misunderstanding of what is and is not possible with ML [2,3]. Here, we aim to provide an accessible, scientifically and technologically accurate portrayal of the current state of ML (often referred to as AI in medical literature) in health and medicine and its potential, using examples of recent research—some from *PLOS Medicine*’s November 2018 Special Issue on Machine Learning in Health and Biomedicine, for which we served as guest editors. We have selected studies that illustrate different ways in which ML may be used and their potential for near-term translational impact.

## ML-assisted diagnosis

Of the myriad opportunities for use of ML in clinical practice, medical imaging workflows are most likely to be impacted in the near term. ML-driven algorithms that automatically process 2- or 3-dimensional image scans to identify clinical signs (e.g., tumors or lesions) or determine likely diagnoses have been published, and some are progressing through regulatory steps toward the market. Many of these use deep learning, a form of ML based on layered representations of variables, referred to as neural networks. To understand how deep learning methods leverage image data to perform recognition tasks, imagine you are entering a dark room and looking for the light switch. From past experience, you have learned to associate light switches with predictable locations within the configuration of a room. Many computer vision–based image processing algorithms, including deep learning, mimic this behavior to identify factors that are associated with the recognition task at hand. Deep learning is especially powerful in its ability to interpret images because of the complexity of the factors it can consider.

The power of deep learning has been most evident within ophthalmology. Recently, Olaf Ronneberger and colleagues applied a two-step process using deep learning to a clinically heterogeneous set of 3-dimensional optical coherence tomography (CT) scans from patients referred to a major United Kingdom eye hospital [4]. They demonstrated performance in making a referral recommendation that reaches or exceeds that of experts on a range of sight-threatening retinal diseases after training on only 14,884 scans. In another effort, IDx, a healthcare automation company, has developed deep learning–based software to be used by health providers who treat patients with diabetes to scan images for signs of diabetic retinopathy [5]. Their cloud-based, autonomous detection software has received regulatory approval by the United States Food and Drug Administration (FDA). With the volume and complexity of diagnostic imaging increasing faster than the availability of human expertise to interpret it (especially in low-resource settings), screening for referable disease or detecting treatable disease in patients who would not otherwise receive eye exams may save both vision and money.

Radiologic diagnoses are also amenable to deep learning–based applications. In a study in *PLOS Medicine*’s Special Issue, Pranav Rajpurkar and colleagues used a deep learning algorithm to detect 14 clinically important pathologies including pneumonia, pleural effusion, pulmonary masses, and nodules in frontal-view chest radiographs with internal performance similar to practicing radiologists [6]. The algorithm, called CheXNeXt, was trained, tuned, and internally validated on partitioned subsets of the National Institutes of Health (NIH) ChestX-ray8 dataset (over 100,0000 chest radiographs from roughly 31,000 patients). The model's performance was compared to that of 9 radiologists (6 board-certified, 3 residents) using a held-out partition of the dataset consisting of images hand-annotated by a panel of cardiothoracic specialist radiologists. At comparable accuracies, the average time to interpret the 420 images in the validation set was substantially longer for the radiologists (240 minutes) than for CheXNeXt (1.5 minutes). The model also localized parts of the image most indicative of each pathology. A tool such as this, though still early in its development, offers a solution to fatigue-based diagnostic error and lack of diagnostic expertise in the many areas of the world where radiologists are not available or are in short supply.

## ML-driven triage and prevention

Prediction to aid preventative efforts is another promising frontier for improving outcomes using ML. For example, in the Special Issue, a study from Kristin Corey and colleagues considered the potential for reducing complications and mortality within 30 days following particular surgeries [7]. Using data from about 88,000 encounters extracted from June 2012 to June 2017, they developed software (Pythia) that incorporates a patient’s age, race, sex, medication, and comorbidity history to determine risk of complications or death post surgery. Overall, postsurgical complication rates were 16.0% for any complication within 30 days and 0.51% for death within 30 days. In a separate validation set of 12,000 encounters, at a threshold selected to have sensitivity of 0.75, Pythia achieves a positive predictive value of 0.35; in other words, 1 in 3 patients flagged by their approach have a postsurgical complication within 30 days. Comparison of Pythia’s scores to scores from The American College of Surgeons (ACS) National Surgical Quality Improvement Program (NSQIP) calculator on a smaller set of 75 encounters found that Pythia identifies higher-risk patients. A tool like Pythia can enable surgeons and referring clinicians to identify high-risk individuals who may require targeted assessments and optimization as part of their preoperative care. For example, a patient with anemia at high risk for a hematological complication such as bleeding may benefit from being put on iron transfused with blood prior to surgery or have medications managed to help mitigate the risk of losing blood during the procedure. The efficacy with which such algorithms can be operationalized to improve clinical adoption is a key question. Unlike in medical imaging applications, here the goal is to augment rather than automate existing workflows. Efforts testing such workflows in sepsis, a leading cause of death and one of the costliest complications, are underway at institutions such as Johns Hopkins and Duke, with the former system beginning to demonstrate benefit [8–10].

In another Special Issue study relevant to prevention, Yizhi Liu and colleagues used real-world clinical refraction data from about 130,000 individuals aged 6 to 20 years derived from electronic medical record (EMR) systems in 8 ophthalmic centers from 2005 to 2015 to predict myopia progression [11]. Myopia has reached epidemic levels among young adults in East and Southeast Asia, affecting an estimated 80%–90% of high school graduates, with approximately 20% of them having high myopia. Various preventative interventions, including atropine eye drops and orthokeratology, have been proposed to control myopia progression; however, these approaches confer significant side effects. Therefore, it is essential to identify those at greatest risk who should undergo targeted therapy. On a large multisite dataset, Liu and colleagues’ approach was able to predict the refraction values and onset of high myopia at 18 years of age as early as 8 years in advance with clinically acceptable performance (the authors considered ±0.75 dioptres clinically acceptable accuracy). This model is now ready for evaluation in prospective studies to determine whether behavioral or clinical interventions can delay progression of myopia among high-risk school-aged children in China.

## Clustering for discovery of disease subtypes

The definitions of diseases and disease subtypes we use today are based largely on the original symptom-based descriptions offered in the 17th century by Sydenham and Linnaeus and the organ-based definitions developed by Osler in the 20th century. It is, however, now possible to move beyond these observational approaches to more data-driven approaches to diagnosis and disease classification. In a series of experiments, Adnan Custovic and colleagues have been pursuing this approach in the context of asthma and allergy. Using unsupervised ML, the group analyzed data from the Manchester Asthma and Allergy Study (MAAS) population-based birth cohort and were able to identify novel phenotypes of childhood atopy [12]. Through further interrogation of this same dataset, the authors have now identified clusters of component-specific immunoglobulin E (IgE) sensitization using network and hierarchical cluster analysis that can help better predict risk of childhood asthma [13]. We believe there are considerable opportunities to employ similar data-driven approaches to aid diagnostic processes in other disease areas, and using ML methods to find new actionable disease subsets will be critical to advance precision medicine [14].

## Reducing medication errors via anomaly detection

Medication errors are responsible for considerable—and potentially preventable—morbidity, mortality, and healthcare costs. These errors can be identified through a variety of means, including expert chart reviews, use of triggers, rules-based approaches to screening EMRs, and significant event audits. However, these approaches are associated with a number of challenges: suboptimal sensitivity and specificity, time consumption, and expense. ML-based anomaly detection techniques begin by developing a probabilistic model of what is likely to occur in a given context by using historical data. Using this model, a new event (e.g., medication given at a particular dose) within a specific context (e.g., individual patient characteristics) is flagged as anomalous if its probability of occurring within that context is very small. MedAware is a commercially available system that uses anomaly detection to generate medication error alerts. In a recent study, Gordon Schiff and colleagues used medical chart review to analyze the validity and clinical utility of these alerts [15] and found that three-quarters of the alerts generated by the screening system were valid according to the charts. Of these validated alerts, the majority (75.0%) were found to be clinically useful in flagging potential medication errors or issues. Such findings indicate that this approach has the potential to be incorporated into clinical use, although Schiff and colleagues do caution that the utility of this system is highly dependent on the quality and comprehensiveness of the underlying data.

## The ML-augmented physician

We have discussed several examples of ML’s potential to transform medical care. However, naive implementation of ML without careful validation can also harm patients and the public. Consider, as an example, a hypothetical effort to predict the risk of emergency hospital admissions using a model trained on past admissions data for patients with various characteristics and symptoms. Actual admissions are often subject to bed availability, the type of insurance an individual is carrying, and reimbursement practices. Whereas this trained model might enable population-level resource planning, attempting to use it for individual-level triage may incorrectly classify an individual as not requiring an admission. To some extent, an ML algorithm can replicate past decisions, including biases around race and sex that may have influenced clinical judgement about the level of care given. “Irrational extrapolation”—the assumption that algorithms trained on an easy-to-obtain set of patients or data will lead to accurate models that act in each patient’s best interest—must be stringently avoided until algorithms can correct for such biases and use clinical data to reason about disease severity and trajectory.

Another pitfall of naive implementation lies in the capacity of ML, and particularly deep learning, to overfit to data—that is, to identify associations in the training dataset that are not truly intrinsic to the clinical prediction and will not be relevant externally [16]. Techniques that leverage causal factors are less likely to be prone to such overfitting (e.g., [17]), and conscientious construction of training datasets and multiple external validation efforts for each trained model can provide some assurance that ML-based models are valid. These developments within computer science, alongside high standards for validation among medical data scientists, are crucial if ML is to benefit future patient care. In parallel, clinicians and clinical researchers who remain aware of successes and needs in the field can be an invaluable force in the optimal development and implementation of these powerful approaches. The new generation of practitioner should not unnecessarily fear ML but rather should learn how to understand, develop, and ultimately leverage it so as to improve patient care.

## Abbreviations

ACS: American College of Surgeons
AI: artificial intelligence
CT: coherence tomography
EMR: electronic medical record
FDA: Food and Drug Administration
IgE: immunoglobulin E
MAAS: Manchester Asthma and Allergy Study
ML: machine learning
NIH: National Institutes of Health
NSQIP: National Surgical Quality Improvement Program
